# The Role of Hospice in Florida Nursing Homes Post-Hurricane Irma

**DOI:** 10.1093/geroni/igab046.1102

**Published:** 2021-12-17

**Authors:** Debra Dobbs, Julianne Skarha, Lily Gordon, Dylan Jester, Lindsay Peterson, David Dosa

**Affiliations:** 1 University of South Florida, School of Aging Studies, University of South Florida, Florida, United States; 2 Brown University, Providence, Rhode Island, United States; 3 Health Services, Policy and Practice, Brown University, Providence, Rhode Island, United States; 4 University of California San Diego, La Jolla, California, United States; 5 University of South Florida, Bradenton, Florida, United States

## Abstract

There is little known about the effect of hospice post-disaster. This study utilized exposure to Hurricane Irma (2017) to evaluate the differential mortality effect of the disaster on Florida NH residents (N=45,882) compared to a control group of residents in the same NHs in 2015 (N=47,690) by hospice status. We also examine the difference in hospice utilization rates post-storm for short- and long-stay (LS) residents. There was an increase in mortality for those in the cohort not on hospice within 90 days in 2017 compared to 2015 (OR= 1.06, 95% CI: 1.01, 1.11). For the rate of hospice enrollment post-storm among residents previously not on hospice, there was an increase among LS residents within 30 days (OR =1.15, 95% CI: 1.02, 1.23) and 90 days (OR= 1.12, 95% CI: 1.05, 1.20). It is important to further examine the increase in the rate of hospice enrollment in LS NH residents post-storm.

